# Self-Coexistence among IEEE 802.22 Networks: Distributed Allocation of Power and Channel

**DOI:** 10.3390/s17122838

**Published:** 2017-12-07

**Authors:** Sayef Azad Sakin, Md. Abdur Razzaque, Mohammad Mehedi Hassan, Atif Alamri, Nguyen H. Tran, Giancarlo Fortino

**Affiliations:** 1 Department of Computer Science and Engineering, University of Dhaka, Dhaka 1000, Bangladesh; sayefsakin@gmail.com (S.A.S.); razzaque@du.ac.bd (M.A.R.); 2 College of Computer and Information Sciences, King Saud University, Riyadh 11543, Saudi Arabia; atif@ksu.edu.sa; 3 Department of Computer Science and Engineering, Kyung Hee University, Gyeonggi-do 17104, Korea; nguyenth@khu.ac.kr; 4 Department of Informatics, Modeling, Electronics, and Systems, University of Calabria, 87036 Arcavacata, Italy; g.fortino@unical.it

**Keywords:** IEEE 802.22, cognitive radio, WRAN, OFDMA, game theory, Nash equilibrium, non-linear optimization, distributed algorithm

## Abstract

Ensuring self-coexistence among IEEE 802.22 networks is a challenging problem owing to opportunistic access of incumbent-free radio resources by users in co-located networks. In this study, we propose a fully-distributed non-cooperative approach to ensure self-coexistence in downlink channels of IEEE 802.22 networks. We formulate the self-coexistence problem as a mixed-integer non-linear optimization problem for maximizing the network data rate, which is an NP-hard one. This work explores a sub-optimal solution by dividing the optimization problem into downlink channel allocation and power assignment sub-problems. Considering fairness, quality of service and minimum interference for customer-premises-equipment, we also develop a greedy algorithm for channel allocation and a non-cooperative game-theoretic framework for near-optimal power allocation. The base stations of networks are treated as players in a game, where they try to increase spectrum utilization by controlling power and reaching a Nash equilibrium point. We further develop a utility function for the game to increase the data rate by minimizing the transmission power and, subsequently, the interference from neighboring networks. A theoretical proof of the uniqueness and existence of the Nash equilibrium has been presented. Performance improvements in terms of data-rate with a degree of fairness compared to a cooperative branch-and-bound-based algorithm and a non-cooperative greedy approach have been shown through simulation studies.

## 1. Introduction

Cognitive radio (CR) [1,2] is the most advanced technology for increasing the spectrum utilization efficiency of many radio users. This technology frontier promises to deal with the spectrum shortage problem of the conventional inflexible frequency allocation policy. Experiential learning helps CR-enabled devices to make intelligent decisions, which facilitate vacant spectrum utilization without disturbing the licensed users. The licensed users are considered as primary users (PUs), and CR-based unlicensed users are called secondary users (SUs). Recently, CR techniques have been used in WSNs (termed CR-WSNs) to overcome the inherent limitations of traditional WSNs [3,4]. CR-WSN allows unlicensed users to access multiple licensed channels opportunistically and gives great advantages to WSNs to increase their communication power and the energy efficiency. A wireless sensor node with cognitive capabilities can provide various new opportunities to develop algorithms, hardware and software that can overcome the limitations of WSNs. Exploiting the present progression in CR-WSN development, it is now possible to mitigate the various issues in terms of spectrum inefficiency in the WSN scenario.

**Figure 1:**
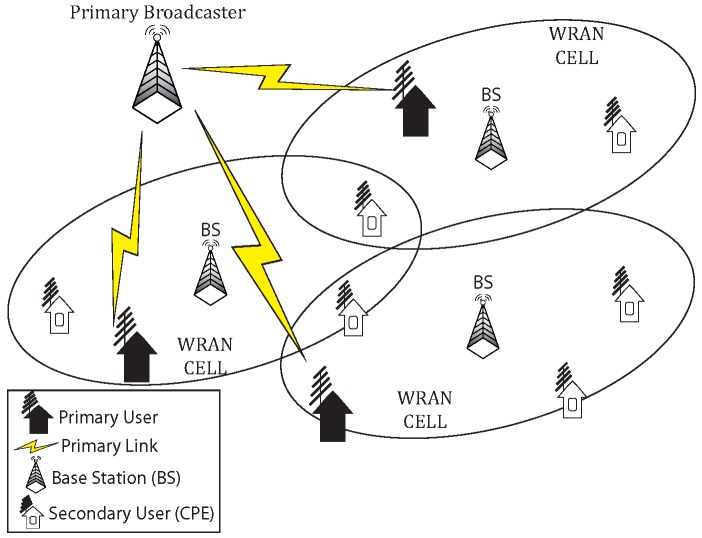
The IEEE 802.22 network architecture. WRAN, wireless regional area network; CPE, customer-premises-equipment.

The IEEE 802.22 wireless regional area network (WRAN) is the first wireless standard based on CR technology [5] that operates over the licensed TV bands (54 MHz–862 MHz) [6]. It is an infrastructure-based WRAN, where a base station (BS) controls stationary wireless subscribers called customer-premises-equipment (CPE) (Figure 1). Each BS forms a single wireless network (CR cell) and manages the resource access and scheduling in its own WRAN. It is used as the base infrastructure network for smaller WSNs. Significant research studies on primary-secondary spectrum etiquette [7,8] efficiently characterize primary incumbent activities in different regulatory domains. The IEEE 802.22 WRAN entities use geolocation databases [9] with spectrum sensing techniques for tracking white spaces. These research studies mostly ignored (or partially covered) the secondary-secondary spectrum etiquette such as maintaining quality-of-service (QoS) or fairness between SUs within co-located CR network cells. Self-coexistence refers to the ability of ensuring interference-free transmissions among neighboring homogeneous CR network cells. The IEEE 802.22 standard proposes time division multiple access (TDMA)-based self-coexistence mechanisms such as dynamic resource renting and offering (DRRO) and adaptive on demand channel contention (AODCC) [5]. Conforming to these self-coexistence mechanisms, the CR networks in densely-populated urban areas are vulnerable to backhaul network downtime, beacon flooding, false QoS demands, prior network knowledge distribution, recursive contention [7,10], etc. Therefore, an autonomous and dynamic channel access among co-located CR network cells is necessary for reducing interference.

Graph coloring-based techniques are used in [11,12] to improve the self-coexistence mechanism for WRAN. A bipartite matching-based channel allocation algorithm was proposed in our previous work [13]. Kaushik et al. [14] propose a joint power and bandwidth allocation in IEEE 802.22-based CR LTE networks. In real life, co-located CR cells can be operated by different service providers, making these centralized approaches obsolete. In [15,16], the authors utilize game theory-based models for autonomous channel scheduling. Even though these models improved channel utilization, they failed to achieve better data rates and fairness among networks. Therefore, an efficient distributed approach for resource allocation (channel, power, etc.) ensuring QoS and fairness of the IEEE 802.22 WRAN is necessary.

In this study, we address the self-coexistence problem in co-located uncoordinated IEEE 802.22 WRANs (Figure 1) for CR-WSNs. The network cells may be partially (or completely) overlapped, and they compete for the spectrum resources and try to find out interference-free spectrum bands from other coexisting network cells. A narrowband interference-free common control channel (CCC) is used for transferring control messages between BSs and CPEs. The objectives are to increase network data rate, minimize global power consumption and gain intra-cell fairness while maintaining QoS. To attain the proposed objectives in multi-cell environment, we formulate a mixed-integer nonlinear optimization problem that enhances the data rate. Then, we decompose the problem into two subproblems, subchannel assignment and power allocation for individual network cells. We also propose a greedy channel assignment algorithm and a game-theoretic power allocation mechanism to solve the above subproblems distributedly. The solution scheme aims to achieve a better channel utilization ratio while minimizing power loss of the BSs. The key contributions of this study are summarized as follows:

- We formulate the self-coexistence problem as a mixed-integer nonlinear optimization problem that maximizes the network data rate by allowing neighboring base stations to allocate opportunistic resources to SUs in a non-interfering manner.
- Owing to the NP-hardness of the formulated problem, we decompose it into two subproblems to achieve near optimal solutions. We develop a greedy subchannel assignment algorithm considering channel gain and interference(s).
- We propose a game-theoretic model and utility function for power allocation in the BSs. Then, we derive the best response function for the BSs based on network data rate and power consumption.
- We provide a theoretical proof of the unique existence of the Nash equilibrium (NE) point and develop distributed algorithms that can guide each BS toward that point.
- The simulation results, experimented on ns-3 [17], show that the proposed resource allocation method outperforms state-of-the-art works in terms of data rate, fairness, convergence cost and power usage.

The rest of the paper is organized as follows. In Section 2, we present a review of existing works on this issue. The network model and problem formulation parts are presented in Section 3. In Section 4, the proposed solution methodology and the game model are described. In Section 5, the performance evaluation of the proposed system through simulation experiments is presented, and this is followed by the conclusion in Section 6.

## 2. Related Works

The fluctuating nature of the spectrum in diverse CR networks produces manifold challenges for spectrum management [10]. Statistical prediction-based models for primary incumbents [8] empower secondary users to adopt spectrum-aware channel assignment techniques for unexploited radio resources [18,19]. Cognitive radio sensor networks (CRSN) [20] harness this spectrum opportunity with channel bonding, channel aggregation, channel assembling and channel width adaptation techniques [18]. Bukhari et al. [21] propose algorithms to find contiguous channels for bonding together in CRSN. These spectrum-sharing techniques can be used in cellular networks based on code-division multiple access (CDMA) or carrier-sense multiple access (CSMA) channels [18]. For more spatial frequency utilization, CDMA is used together with orthogonal frequency-division multiplexing (OFDMA) in which one aggregated CDMA channel is further divided into multiple subchannels [5,22,23]. OFDMA enables co-located networks to operate over same channel in different subchannels as long as interference among those resides below a certain signal-to-noise-ratio (SINR) level [22,24]. This imposes new research challenges for self-coexistence in homogeneous wireless CR networks.

The self-coexistence mechanism in the IEEE 802.22 standard uses the self-coexistence window (SCW) and the co-existence beacon protocol (CBP) [5] for implementing beacon transmissions among network cells. The IEEE 802.22 inter-BS coexistence mechanism [5] consists of four stages: spectrum etiquette, interference-free scheduling, DRRO and AODCC. Spectrum etiquette is conducted where BSs try to locally find channels that their neighboring BSs do not use. When there is a lack of available channels, BSs will conduct interference-free scheduling, in which they share the same channel by scheduling traffic in a non-interfering manner.

In DRRO, the IEEE 802.22 standard defines broadcasting beacon messages among renter and offerer BSs, and channel sharing is conducted with proper acknowledgment messages between them. In AODCC, the renter BS becomes the contention source and randomly selects a channel contention number (CCN) from a uniformly-distributed range. Contention sources broadcast the CCN toward the contention destination BSs, which choose source BSs with a greater CCN value for the contending channels. These TDMA-based self-coexistence mechanisms cannot ensure resource scheduling for highly co-located CR cells. Adapting an orthogonal frequency division multiple access (OFDMA) for interference-free scheduling creates a new problem domain for resource demands. Recent research trends highly follow the self-coexistence techniques on OFDMA channels [25,26].

There have been several research works conducted to improve the self-coexistence mechanism of the IEEE 802.22 standard. In [11,27,28], the authors address the self-coexistence problem by a graph coloring perspective. They model the network using graph theory by representing network cells, interference among them and channels with nodes, edges and colors, respectively. By using different graph coloring techniques, they complete the channel assignment to each network cell. The authors in [29] use the state-vector (set of networks allowed to transmit over a channel) and recursively calculate the transmission probability for all possible state-vector combinations. Co-located network cells choose a coordinator among them, which caries over the assignment process based on the calculated probability. These research works are highly dependent on a centralized spectrum manager and require complete knowledge of entire networks for graph formation. Greedy algorithms are developed in [30] to solve the channel allocation problem using both cooperative and non-cooperative methods. While the cooperative approach exploits the conventional graph coloring with the enhancement of node sub-grouping, the non-cooperative approach uses a backoff mechanism for channel scheduling. The authors in [31] propose an artificial intelligence-based regression model for channel association among BSs. This work fails to provide definite applicability and a power control mechanism in the cognitive environment particularly for the IEEE 802.22 networks.

Sengupta et al. [15,32] propose distributed game-theoretic solutions, in which network cells play a non-cooperative game, trying to reach an NE by channel switching. They model the game as a modified minority game (MMG) with a combined strategy. Their goal is to increase throughput by minimizing false detection. A Similar non-cooperative game approach is adopted in [33] with refined strategies. They also improve and analyze the expected cost function for channel switching. An inter-BS coexistence technique for IEEE 802.22 is developed in [34] based on resource renting, offering and contention. In this work, the authors update the conventional resource renting-offering algorithm with a unique resource-sharing game model and explore different renter-acquirer scenarios. In [16], the authors use a physical interference model to identify overlapping network cells and model channel assignment as a potential game (PG). Their goal is to achieve an increased signal-to-noise-ratio (SINR) value throughout the network. These research works highly focus on channel scheduling while ignoring power assignment in different channels.

The downlink channel allocation problem has been focused on in [24,35,36,37]. Kibria et al. [35] propose a modified OFDM modulation for downlink resource scheduling in machine-to-machine (M2M) network [38]. Their greedy subchannel scheduling is limited by their proposed modulation technique for the M2M network. The authors of [36] only consider a single cell network and ignore the co-located network environment. Choi et al. [24] consider general CR networks, where neighboring cells use a partial frequency reuse scheme to access channels. In [37], the authors propose a channel/power allocation with global and local knowledge of active CPEs. Although the authors of [24,37] provide embedded primary incumbent parameters, their methodology lacks fairness of assignments and requires direct cooperation of PUs.

A branch-and-bound (B&B) algorithm for IEEE 802.22-based LTE networks has been developed in a queue-based control (QBC) [14], where resource allocation is controlled by the queue size of nodes following their packet arrival probabilities. They achieve optimal power and resource block assignment for each mobile user, trading execution time and end-to-end packet delay.

The existing works are highly dependent on a centralized spectrum manager and a backbone network that connects BSs to each other. Co-located networks operated by different service providers cannot connect to each other and share information between them. Again, they require collecting the entire network information in a centralized place for running the optimization. Gathering information in a centralized place causes overhead in the overall execution and leads to incorrect scheduling owing to obsolete information and, in some cases, is not realistic. Therefore, an autonomous and distributed approach for self-coexistence without the cooperation of PUs is required to ensure user QoS and fairness with minimum power consumption.

## 3. Problem Definition

### 3.1. System Model

We consider the IEEE 802.22 WRAN standard [39], where *N* number of cognitive network cells coexist, represented by a set N≡{1,2,⋯,N}. The total frequency spectrum band (6 MHz TV band) is divided into *K* separate orthogonal subchannels with equal bandwidth *B*. In each network cell, there is a BS serving each stationary CPE opportunistically with one of the *K* subchannels. There is a total of *C* CPEs in the area, where each CPE c∈C≡{1,⋯,C} can initiate one or more sessions. Here, a session is defined as a unique connection demand of a CPE, which will be fulfilled by the BSs. Each CPE c∈C in a cell n∈N can maintain multiple sessions at a time, and each session $s\in S_{c}^{n}\equiv \left\{1,\cdots ,S_{c}^{n}\right\}$ can operate on any of the channels. Each subchannel k∈K≡{1,⋯,K} will be assigned to no more than one session in the same network cell at a time. Because of the high possibility of finding a CCC in a confined regional area [40], it is considered that contention-free usage of CCC is regulated by a suitable mechanism [41,42].

In this study, we only consider the downlink transmission (from BS to CPE) in a multi-cell OFDMA network, as shown in Figure 1. A BS searches for incumbent-free subchannels and assigns downlink subchannels to the CPE. The BSs may assign multiple sessions to a CPE, which requires more frequency bandwidth. A list of major mathematical symbols used in this study is given in Table 1.

### 3.2. Downlink Channel Allocation and Power Control

Each CPE periodically senses the available subchannels and shares the sensing results with its BS. For any power allocation vector P, the total interference from all users measured across a session *s* on a subchannel *k* at BS *n* is given by:

(1) $$I_{s,k}^{n}=I_{c,k}^{PU}+\sum_{a=1,a\neq n}^{N}h_{s,k}^{a}\;p_{k}^{a},\;\forall k\in K,\forall s\in S_{c}^{n},\forall c\in C,$$

where *n* is the BS to which the session of the CPE is registered. Now, we can calculate the achieved SINR $\sigma_{s,k}^{n}$ of a session *s* in subchannel *k* at BS *n* as follows:

(2) $$\sigma_{s,k}^{n}=\frac{h_{s,k}^{n}\;p_{k}^{n}}{I_{s,k}^{n}+\eta_{0}}.$$

Each CPE sends this sensing information $\left(\sigma_{s,k}^{n}\right)$ to its associated BS, which then calculates the achievable data rate using Shannon’s capacity formula as follows:

(3) $$q_{s,k}^{n}=B\log_{2}\left(1+\sigma_{s,k}^{n}\right).$$

A BS also calculates the achievable maximum data rate for each session $s\in S^{n}$ if it uses the maximum transmission power $p_{\max}^{n}$ in channel *k*.

(4) $$Q_{s,k}^{n}=B\log_{2}\left(1+\frac{h_{s,k}^{n}\;p_{\max}^{n}}{I_{s,k}^{n}+\eta_{0}}\right).$$

Now, we generate the relative data rate as:

(5) $$R_{s,k}^{n}=\alpha \times \frac{q_{s,k}^{n}}{Q_{s,k}^{n}}-\left(1-\alpha \right)\times \frac{p_{k}^{n}}{p_{\max}^{n}}.$$

The relative data rate $R_{s,k}^{n}$ represents the relationship between the power and data rate in each session. As our objective is to maximize the data rate using the least transmission power as possible, we take the difference of the normalized power from the normalized value of the data rate. Here, α is a control parameter, generally set by the network designer in each BS. This parameter is fixed for each BS, and it helps to provide control over the power and data rate. The relative data rate in Equation (5) is the difference between the data rate ratio and power ratio multiplied by α and (1−α), respectively. α>0.5 will reduce the power ratio more than the data rate ratio, which leads to more positive values. Therefore, the rate of change of the data rate ratio will be higher than that of the power ratio. On the other hand, α<0.5 will increase the power ratio more than the data rate ratio, which leads to more negative values. Therefore, the rate of change of the data rate ratio will be lower than that of the power ratio.

Now, our combined problem of downlink subchannel allocation and power control boils down to maximizing this relative data rate for all sessions and subchannels in all networks. Thus, the problem is formulated as follows:

(6) $$\begin{array}{ccc} \max_{D,P} & \sum_{n\in N}\sum_{s\in S^{n}}\sum_{k\in K}R_{s,k}^{n}d_{s,k}\; & \end{array}$$

(7) $$\begin{array}{ccc} s.t.\; & 0\leq p_{k}^{n}\leq \sum_{s\in S^{n}}d_{s,k}p_{\max}^{n}, & \;\forall k\in K,\;\forall n\in N \end{array}$$

(8) $$\begin{array}{cc} \sum_{k\in K}\sum_{s\in S^{n}}d_{s,k}\;p_{k}^{n}\leq p_{\max}^{n}, & \;\forall n\in N \end{array}$$

(9) $$\begin{array}{cc} \sum_{k\in K}d_{s,k}q_{s,k}^{n}\geq \theta_{s}\sum_{k\in K}d_{s,k}, & \;\forall s\in S^{n},\forall n\in N \end{array}$$

(10) $$\begin{array}{cc} \sum_{k\in K}d_{s,k}\leq 1, & \;\forall s\in S^{n},\;\forall n\in N \end{array}$$

(11) $$\begin{array}{cc} \sum_{s\in S^{n}}d_{s,k}\leq 1, & \;\forall k\in K,\;\forall n\in N \end{array}$$

(12) $$\begin{array}{cc} d_{s,k}\in \left\{0,1\right\}, & \;\forall k\in K,\;\forall s\in S \end{array}$$

(13) $$\begin{array}{cc} \sum_{s\in S^{n}}\sum_{k\in K}d_{s,k}\leq K, & \;\forall n\in N \end{array}$$

Here, BSs from all network cells gather relative data rate values $R_{s,k}^{n}$ from each session s∈S for each subchannel k∈K. The objective function in Equation (6) is to maximize the total sum of the relative data rates for all sessions and all channels. That is, it always tries to choose the set of sessions that achieves the maximum data rate with minimal power increment. Note that a CPE residing in interfering zones may produce a negative relative data rate value because the BS has to use very high power for it to achieve the required SINR. Thus, the objective function would eventually remove such CPE from the optimal subchannel allocation.

The constraints of the objective function are given as Equations (7)–(13). Equations (7) and (8) represent the transmission power allocation limits for the subchannels in a BS. Equation (7) provides that the transmission power of a BS in a subchannel is bounded by its maximum transmission power. We take a separate constant $p_{\max}^{n}$ for each BS n∈N without loss of generality even though the maximum transmission power can be the same for all BSs. Equation (8) limits the total power allocated to all sessions in different subchannels by the maximum power of a BS.

Equation (9) ensures the user QoS for each session by measuring the expected data rate. Equations (10) and (11) are the subchannel assignment constraints, i.e., one subchannel can be assigned to at most one session under a BS and vice versa. Equation (12) is a domain constraint for subchannel allocation. The maximum number of sessions that can be allocated is limited by the total number of available subchannels in the network based on Equation (13).

We observe that the above optimization problem belongs to a class of mixed-integer nonlinear optimization problems. For single-channel networks and small number of CPEs, this problem can be solved in polynomial time by converting it into a sequence of approximating linear programs [43]. However, for densely co-located CPEs in networks with a large number of channels, this problem becomes intractable. Furthermore, we require a global coordinator for accommodating and running the optimization solver on behalf of all the network cells. It is impractical to choose a global coordinator in our assumed network environment as different networks are run by dissimilar service providers. It is also assumed that the neighboring networks do not exchange information. In the real-world environment, it is fairly common to deploy different networks by service providers in co-located areas. Therefore, a distributed approach is urgently required to solve this NP-hard problem.

## 4. Distributed Solution for Self-Coexistence

We propose a distributed approach to solve the mixed-integer nonlinear optimization problem expressed in Equation (6). The proposed system aims to achieve the following goals: (i) the transmission power should be allocated such that both inter-cell and PU interferences are minimized; (ii) channels should be efficiently allocated to increase the data rate; (iii) proportional fairness should be achieved by all CPEs within a cell; and (iv) the user QoS requirement should be achieved for all CPEs. Thus, we develop a distributed self-coexistence and non-cooperative power allocation game (DSPG) that divides the NP-hard problem of Equation (6) into two subproblems: greedy subchannel assignment and non-cooperative game for power allocation. We also provide a simultaneous iterative water-filling algorithm, which harnesses the backoff timer approach to synchronize the independent BSs.

### 4.1. Greedy Subchannel Assignment

We use a greedy approach to assign a subchannel to a CPE by giving each CPE c∈C a certain weight, calculated as follows:

(14) $$w_{c,k}=\frac{h_{s,k}^{n}\;{\tilde{p}}_{k}^{n}}{I_{s,k}^{n}+\eta_{0}},\;\forall k\in K,\;\forall s\in S_{c}^{n},$$

where ${\tilde{p}}_{k}^{n}$ is the assigned power in channel k∈K for a beacon message. For simplicity, we divide the total transmission power equally among all subchannels for the beacon signals. Note that we will obtain the same channel gain $h_{s,k}^{n}$ for each CPE c∈C in a particular subchannel k∈K, because the CPEs are stationary. Again, before any subchannel assignment, a BS does not have any assigned session to any CPE. Therefore, after collecting the sensing results from the CPEs in response to a beacon message, the BS initializes $S_{c}^{n}=1,\;\forall c\in C$. The CPEs located beyond the sensing region of the BS will have zero weight (see Equation (14)), as there will be no response signal for them.

After calculating the weights of the CPEs in different subchannels, a BS n∈N creates a subset $C^{n}\subset C$ as follows:

(15) $$C^{n}=\left\{c\in C|\sum_{k\in K}w_{c,k}>0\right\},\;\forall n\in N.$$

Note that Equation (15) helps a BS to find out active CPEs associated with it. It is assumed that the BSs store subchannel assignment statistics for their CPEs. A BS then calculates the CPE metric as follows:

(16) $$W_{c,k}=w_{c,k}\times \frac{2\sum_{i=1}^{T^{n}}y_{c}^{i}i}{T^{n}\left(T^{n}+1\right)},\;\forall k\in K,\;\forall c\in C^{n},$$

where $T^{n}$ is the total number of historical channel usage information stored for each CPE in BS n∈N and $y_{c}^{i}$ is a Boolean variable indicating whether a CPE $c\in C^{n}$ is assigned to any subchannel at the *i*-th step or not. Let us redefine the *i*-th subchannel assignment matrix instance as $D^{i}=\left\{d_{s,k}^{i}\right\}$ where $1\leq i\leq T^{n}$ and:

(17) $$y_{c}^{i}=\left\{\begin{array}{cc} 1, & \mathrm{if}\;\sum_{s\in S_{c}}d_{s,k}^{i}=0,\forall c\in C^{n}.\\ 0, & \mathrm{otherwise}. \end{array}\right.$$

Here, i=1 represents the current assignment step and $y_{c}^{1}=1$, because no subchannel assignment has been made for the current step. Consecutively, i=2 is the previous subchannel assignment step, i=3 is the one before that, and so on.

A high value of the system control parameter $T^{n}$ takes more historical experiences and thus achieves better results. Note that $w_{c,k}$ represents the subchannel perception of a CPE in terms of SINR. Choosing a CPE based only on $w_{c,k}$ leads to always selecting CPEs with high SINR values, depriving the ones with low values. Because our network environment is composed of high CPE density with a small number of available subchannels, it is more likely that some CPEs will not obtain any subchannel. The term CPE metric $\left(W_{c,k}\right)$ is introduced to address this type of scenario. The CPE metric is just a statistical measurement of any CPEs subchannel acquisition history. With the help of this CPE metric, the low SINR-valued CPEs will be able to access a subchannel sometimes; thus, no CPE will be deprived of network resources.

**Figure 2:**
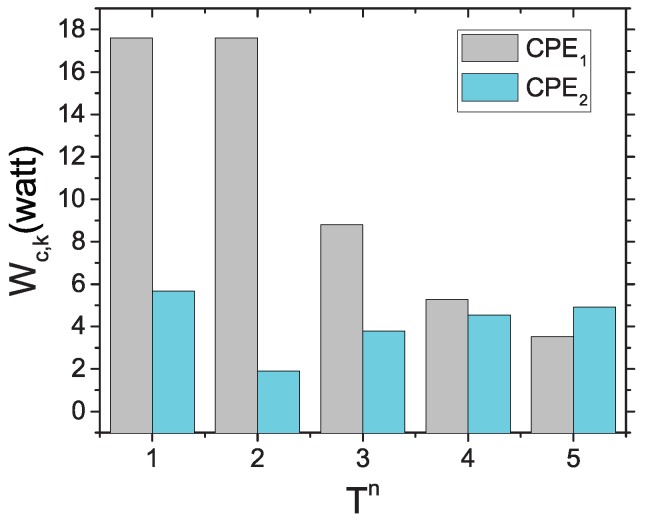
Effect of $T^{n}$ over the CPE-metric.

Let us analyze how historical subchannel usage information helps to improve fairness in subchannel assignment. We present a comparison of CPE metric values for different $T^{n}$ parameters in Figure 2. In this figure, suppose that two CPEs, namely $\left\{CPE_{1},CPE_{2}\right\}\in C^{n}$, are competing over subchannel k∈K. Consider that $CPE_{1}$ obtained subchannel access for $2<T^{n}\leq 5$, while $CPE_{2}$ obtained subchannel access for $T^{n}=2$. For $T^{n}=1$, we obtain the direct SINR values for both CPEs as CPE metrics. When $T^{n}\leq 4,\;CPE_{1}$ has a high CPE metric value; thus, $CPE_{1}$ will be selected for *k*. On the other hand, $T^{n}=5$ provides a high CPE metric value for $CPE_{2}$, which will be selected for subchannel *k*. It helps to clarify that a CPE having the subchannel access for a longer period of time will be penalized more than other CPEs. Thus, the subchannel winning chance will become lower, and a deprived CPE will have a greater chance over the subchannel.

**Figure 3:**
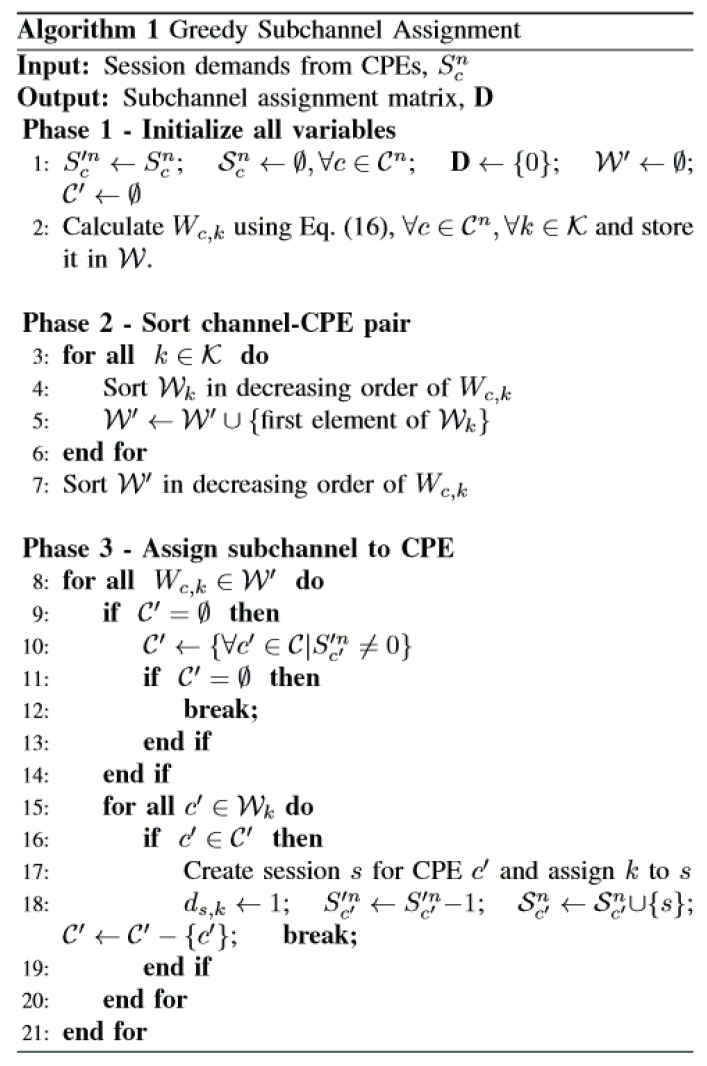
Greedy subchannel assignment.

Algorithm 1 in Figure 3 shows our subchannel assignment procedure for each CPE. We take session demands from CPEs as input, initialize the required variables and calculate $W_{c,k}$ using Equation (16) in Phase 1. We then sort the CPEs and channels in decreasing order of their $W_{c,k}$ values. After completing Phase 2, we have the optimum CPE for each channel in $W^{\prime }$. At the beginning of Phase 3, we create a subset of CPEs having session demands. Then, we assign the best subchannels mapped from $W^{\prime }$ to associated CPEs. The assignment procedure continues until an available subchannel and an unassigned CPE having a session demand exist.

**Figure 4:**
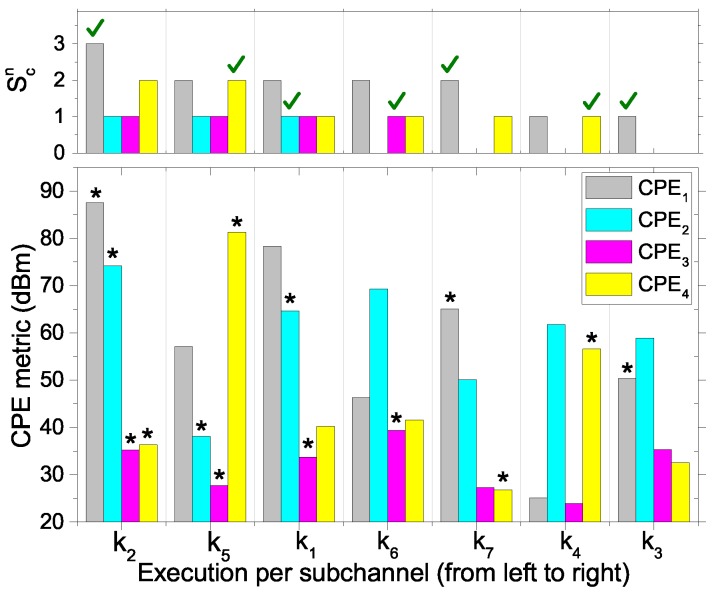
Step-by-step execution of Phase 3 in Algorithm 1.

The following is an illustrative example of the operation of Algorithm 1 in Figure 3. Consider a network n∈N that has seven available subchannels $K=\{k_{1},k_{2},k_{3},k_{4},k_{5},k_{6},k_{7}\}$ and four CPEs $C^{n}=\left\{CPE_{1},CPE_{2},CPE_{3},CPE_{4}\right\}$ having session demand $S^{n}=\left\{3,1,1,2\right\}$. In Figure 4, we present the step-by-step execution of Phase 3 in Algorithm 1. The bottom bars represent CPE metric values in dBm and $T^{n}=1$; therefore, the CPE metric is equal to the SINR value of each CPE in each subchannel. CPEs in the list $C^{\prime }$ are marked with the asterisk (‘*’) sign over the bars. The top bars show current session demand $\left(S_{c}^{n}\right)$ for each CPE. The selected CPEs are marked with a tick (‘√’) sign over the bars. The execution step progresses from left to right over the sorted (by CPE metric value) subchannel list. For subchannel $\left(k_{2}\right)$, all the CPEs are in $C^{\prime }$, and $CPE_{1}$ has a higher CPE metric value with a session demand. Therefore, $CPE_{1}$ is selected for subchannel $k_{2}$, and its session demand is deducted by one in the following step. After the fourth step, $C^{\prime }$ becomes empty, and it is refilled with CPEs $\left\{CPE_{1},CPE_{4}\right\}$ having remaining session demands. The execution stops when no unassigned subchannel or CPE having session demand is left.

### 4.2. Non-Cooperative Game for Power Allocation

Based on the assigned subchannels, each network tries to find out the optimal power allocation for the CPEs. Owing to the distributed nature of the network environment, individually, each BS has to estimate its power for the selected subchannels. We leverage the non-cooperative game theoretic approach [25,44] to characterize the multi-cell power allocation problem. As a player in the game, each BS competes for gaining the optimal relative throughput with different power allocation strategies.

Each BS n∈N participates in the game defined as $G(N,P,\left\{U^{n}\right\})$. Here, $P=\{P^{1},P^{2},\cdots ,P^{N}\}$ is the power allocation matrix, $P^{n}={\left\{p_{k}^{n}\right\}}_{K\times 1}$, where $p_{k}^{n}$ represents the transmission power level of BS n∈N in subchannel k∈K. A BS will participate in the game for a subchannel k∈K if it does not sense any PU signal on the channel and $\sigma_{s,k}^{n}\geq \gamma_{s},\;s\in S^{n}$. This constraint is added to remove hidden incumbent problems. During data transmission, if the condition $\sigma_{s,k}^{n}\geq \gamma_{s}$ fails or the BS senses a PU signal on subchannel *k*, it restrains all transmissions of CPEs and resets all its parameters and its channel allocation procedure. After reaching an NE point, the corresponding transmission power values are assigned to the subchannels.

#### 4.2.1. Game Model and Utility Function

As stated above, each BS n∈N, as a player of the game, independently maximizes its own objectives, as defined in Equation (6). Therefore, the utility function corresponding to the objective in Equation (6) is defined as follows:

(18) $$U^{n}\left(P\right)=\sum_{s\in S^{n}}\sum_{k\in K}R_{s,k}^{n}\left(P\right).$$

If any session experiences high interferences, then the BS power for the session should be increased in order to allow the session to achieve the required minimum data rate. On the other hand, sessions with low interference would gain a better data rate for minimum possible power. In each game iteration, a BS tries to maximize its utility by allocating optimal power for its sessions.

The strategy profile of a BS is denoted by $P=\{P^{n},P^{-n}\}$, where $P^{-n}=\left\{P^{1},P^{2},\cdots ,P^{n-1},P^{n+1},\cdots ,P^{N}\right\}$. The payoff of player *n* for choosing strategy profile P is $U^{n}\left(P^{n},P^{-n}\right)$. Let $P^{*}=\left\{P^{n,*},P^{-n,*}\right\}$ refer to the strategy profile in any NE point; then we have:

(19) $$U^{n}\left(P^{n,*},P^{-n,*}\right)\geq U^{n}\left(P^{n},P^{-n,*}\right).$$

Given the power-based utility function in Equation (18), each BS n∈N selects its power vector $P^{n}$ to maximize its $U^{n}\left(P^{n},P^{-n}\right)$. Because we set $p_{k}^{n}=0$, where $d_{s,k}=0,\forall s\in S^{n}$, we need to determine the value of $p_{k}^{n},\forall s\in S^{n}$ only, where $d_{s,k}=1$. Again, we are calculating the assigned power value after the subchannel assignment using Algorithm 1 in Figure 3, where we will only consider the sessions $s\in S^{n}$, which have been selected for the downlink transmissions in channel k∈K, i.e., $d_{s,k}=1$. By eliminating $d_{s,k}$, the non-cooperative game $G(N,P,\left\{U^{n}\right\})$ can be formulated as:

(20) $$\begin{array}{c} \max_{P^{n}}U^{n}\left(P^{n},P^{-n}\right) \end{array}$$

(21) $$\begin{array}{cc} s.t.\; & p_{k}^{n}\geq 0,\;\forall k\in K \end{array}$$

(22) $$\begin{array}{c} \;\sum_{k\in K}p_{k}^{n}\leq p_{\max}^{n} \end{array}$$

(23) $$\begin{array}{c} \;\sum_{k\in K}q_{s,k}^{n}\geq \theta_{s},\;\forall s\in S^{n} \end{array}$$

**Proposition** **1.** *For the non-cooperative power allocation game presented in Equation (20), a BS n∈N will update its power by the following best response:*

(24) $${\mathrm{BR}}^{n}\left(P^{-n}\right)=P^{n}={\left\{p_{k}^{n*}\right\}}_{K\times 1}$$

*where:*

$$p_{k}^{n*}={\left[\frac{\left(\phi_{1,k}^{n}+\lambda_{3,k}^{n*}\right)B\log_{2}^{e}}{\phi_{2}^{n}-\lambda_{1,k}^{n*}+\lambda_{2}^{n*}}-\frac{1}{\xi_{s,k}^{n}}\right]}^{+}$$

*and:*

$$\phi_{1,k}^{n}=\frac{\alpha }{Q_{s,k}^{n}},\phi_{2}^{n}=\frac{\left(1-\alpha \right)}{p_{\max}^{n}},$$

$$\xi_{s,k}^{n}=\frac{h_{s,k}^{n}}{I_{s,k}^{n}+\eta_{0}}\;\forall n\in N,\forall k\in K.$$

Here, all optimal Lagrange multipliers $\lambda_{1}^{n*},\lambda_{2}^{n*}$ and $\lambda_{3}^{n*}$ can be found (e.g., using bisection method) to satisfy:

$$\sum_{k\in K}{\left[\frac{\left(\phi_{1,k}^{n}+\lambda_{3,k}^{n*}\right)B\log_{2}^{e}}{\phi_{2}^{n}-\lambda_{1,k}^{n*}+\lambda_{2}^{n*}}-\frac{1}{\xi_{s,k}^{n}}\right]}^{+}=p_{\max}^{n}$$

We use the Karush–Kuhn–Tucker (KKT) [43] condition to obtain the above equations. The detailed proof is provided in Appendix A.

The proposition provides us a way of determining the power level of each subchannel for a BS. The second part of the best response is the inverse of SINR. Therefore, it is a water-filling allocation with the water level determined by the first part of the best response function [45]. In the following subsection, we provide the necessary conditions for the convergence of the water-filling solution, i.e., the existence and uniqueness of the NE.

#### 4.2.2. Existence and Uniqueness of the Nash Equilibrium

We have formulated a non-cooperative power allocation game by maximizing the social welfare and also provide the best response for each BS for participating in the game. Let us now prove the existence and uniqueness of the NE.

**Theorem** **1.** There exists an NE of the game $G(N,P,\left\{U^{n}\right\})$, defined in Section 4.2.

**Proof.**  According to the fixed point theorem [46], the following two conditions must be satisfied for the existence of NE in a game [44]:

- The strategy space P is a non-empty, compact and convex subset of a particular Euclidean space.
- The payoff function $U^{n},\;n\in N$ is continuous in P and quasi-concave in $P^{n}$.

Here, the strategy space is a set of power values. In a utility function, we consider the assigned transmission power in channels as the control variable, which is bounded by zero and $p_{\max}^{n}$. Hence, it is definitely non-empty, closed and bounded, i.e., a compact and convex set of $P^{n}$.

According to Equation (18), $U^{n}$ is a linear combination of $p_{k}^{n}$ in $P^{n}$, and hence, $U^{n}$ is continuous in P. Furthermore, it is trivial to show that $U^{n}$ is also concave on its strategy set. ☐

Now, we provide the required conditions for the uniqueness of the NE.

**Theorem** **2.** *If*

(25) $$\max_{n}\left\{\frac{|X_{k}^{n}|}{h_{s,k}^{n}}\sum_{a\neq n}h_{s,k}^{a}\right\}<1,\forall k\in K$$

*where:*

$$X_{k}^{n}=\frac{\alpha p_{\max}^{n}{\left(\xi_{k}^{n}\right)}^{2}{\left(\log_{2}^{e}\right)}^{2}}{\left(\phi_{2}^{n}-\lambda_{1,k}^{n*}+\lambda_{2}^{n*}\right){\left(\log_{2}\left(1+\xi_{k}^{n}p_{\max}^{n}\right)\right)}^{2}\left(1+\xi_{k}^{n}p_{\max}^{n}\right)}-1$$

*for the non-cooperative game $G(N,P,\left\{U^{n}\right\})$; then:*

*1.*  The NE is unique.
*2.*  Starting from any random point, the best response converges to the unique equilibrium in successive iterations.

The key concept of the proof is using the contraction mapping theorem [47] to verify that a particular norm of the Jacobian J is less than one. We explore the value of X and provide the detailed proof in Appendix B.

It should be noted that Equation (25) provides the required condition for the uniqueness of the NE. For a particular CPE, the sum of channel gain from all other interfering BSs on channel *k* must be less than the channel gain of its associated BS on that channel. This should also restrict the position of the CPEs to be not far away from the associated BS. The interference from neighboring BSs and PUs is captured by the value of X. The interesting fact is that, as long as the CPE resides in a less interfered region, the uniqueness always holds. The increment of interference may be controlled by the co-efficient value α, which can be set by the network designer for each BS.

### 4.3. Iterative Water-Filling Power Allocation

**Figure 5:**
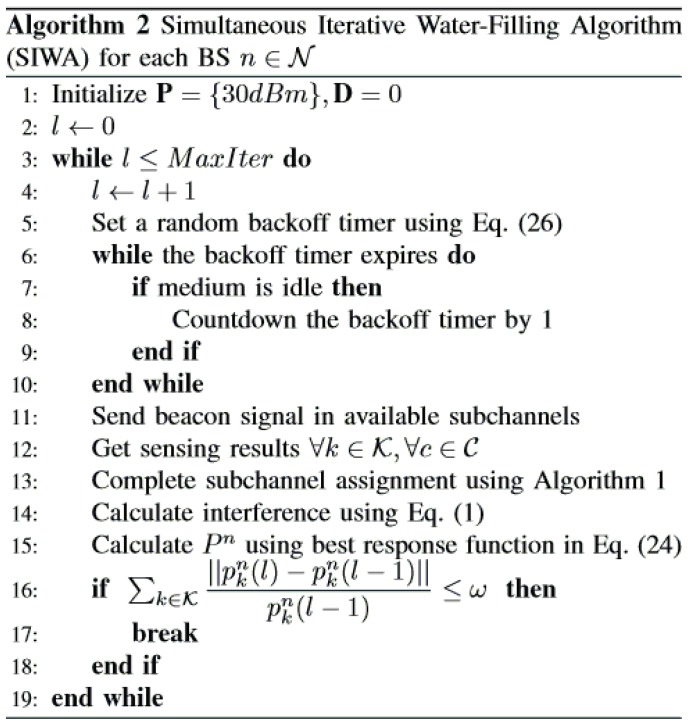
Simultaneous iterative water-filling algorithm (SIWA) for each BS n∈N.

In Section 4.1 and Section 4.2, we have developed two distributed and non-cooperative approaches for solving the optimization problem in Equation (6). We have showed that the best response function is a water-filling solution for each BS. We have also proven the uniqueness and existence of the NE. In this section, we present a simultaneous iterative water-filling algorithm (SIWA) (Algorithm 2 in Figure 5), which exploits the aforementioned approaches to solve the power and channel allocation problem in coexisting WRANs.

To save energy, each BS independently wakes up from the sleep state and starts the sensing and channel-power allocation process. It is necessary to maintain the time-synchronization of the BSs with each other and ensure that no two BSs run the process at the same time. There are several research works [11,16,48] on finding the perfect time interval to ensure excellent synchronization among CR networks. We use [16] for defining the backoff window $b^{n},\;n\in N$ as follows:

(26) $$b^{n}=\left[F^{n},F^{n}+\hat{n}\right]$$

where:

$$F^{n}=\min_{k\in K}\frac{I_{s,k}^{n}}{\gamma_{s}},\;\forall s\in S_{c}^{n}.$$

Here, $\hat{n}$ is the number of neighboring networks that a BS *n* senses. Thus, the backoff window size, as defined in Equation (26), reflects the amount of interference faced by a BS *n* on the chosen subchannels. A BS experiencing a high interference level will try to change its subchannel more frequently. A backoff counter value is chosen randomly from the backoff window and is decreased by one at each time slot when the medium is found free. When the backoff timer of a BS reaches zero, it wakes up and continues the resource allocation process. In the next iteration, the backoff window and timer are reset. The above process provides the BSs with their fair participation in the game so that they would earn a weighted share of the resources for their CPEs. It also facilitates the joining process of the CPEs residing in the overlapped regions of multiple BSs.

In Algorithm 2 in Figure 5 from Lines 6–10, the backoff mechanism is presented. After waking up, each BS collects sensing results from its CPEs (Lines 11 and 12) by broadcasting a beacon signal. The CPEs send the channel sensing information to the BSs when they receive the beacon signal from the BSs. Time and channel synchronization between BS and CPE is done by following the regulatory protocol of the IEEE 802.22 standard. Therefore, a synchronized CPE will always get a beacon signal sent by the BSs in the downstream subframe. A BS experiencing a high interference level will try to change its channel more frequently. As a result, the BS sends a beacon signal, and consequently, the CPEs send back sensing results more frequently. The sensing result sent to the BS is a two-dimensional array of a BS subchannel, along with the PU interference in each subchannel. Each element of this array contains the sensed signal (multiplication of power and channel gain) for each BS and each subchannel. With these sensing results, a BS can find its new power value using the best response in Equation (24).

After receiving the sensing results, the BS completes the channel assignment procedure using Algorithm 1 in Figure 3. With the power allocation values in the previous iteration, the BS calculates the interference using Equation (1) in Line 14. It computes the power values for the current iteration using the best response given in Equation (24) (Line 15). Line 16 in Algorithm 2 checks whether the system reaches an equilibrium state. If the condition is met, we stop the iteration. The value of ω ranges as 0<ω<<1; this helps the algorithm to tune the equilibrium condition. We obtain more accurate results if ω gets closer to zero. The variable MaxIter is the upper bound of the iteration counts to avoid infinity loops. The convergence of the simultaneous iterative water-filling approach for a Gaussian frequency-selective interference channel is proven in [45]. Here, in a similar environment, SIWA water fills each subchannel power to the designated water level determined by the first part of the best response. Hence, our SIWA will eventually converge to a unique NE with successive iterations.

We now estimate the asymptotic complexity of our proposed algorithm for a single iteration. At first, we calculate the complexity of Algorithm 1 in Figure 3. In Lines 3–6, we sort CPEs for each subchannel, which costs O(KClog(C)). Sorting selected subchannels in Line 7 has a cost of O(Klog(K)). Hence, the total complexity in Phase 2 is O(KClog(C)+Klog(K))=O(K(Clog(C)+log(K))). In Phase 3, we prepare a list of CPEs having session demands and assign them the best matched subchannels (high CPE metric value). We search through the CPE list for each subchannel, assign a CPE with session demand to that subchannel and remove it from the session demand list. For the worst case scenario, the first subchannel will get the first CPE; the second subchannel will get the second CPE (if the first one was removed from the session demand list), and so on. This process is done once for each CPE in the session demand list. Since there can be at most *C* items in this session demand list, subchannel assignment for a session demand list has complexity of $O\left(C\left(C+1\right)/2\right)\approx O\left(C^{2}\right)$. If there is an unassigned subchannel and the session demand list becomes empty, it is refilled with CPEs having more session demands. Note that there will be $min(K,S^{n})$ assignment tasks needed in Phase 3. Since one iteration over the session demand list costs $O(C^{2})$, which completes *C* assignments among $min(K,S^{n})$, to complete all the assignments, the total number of iterations needed over the session demand list is at most min $(K,S^{n})/C$. Therefore, the total complexity of Phase 3 is $O\left(\left\lceil \frac{\min(K,S^{n})}{C}\right\rceil C^{2}\right)$. The best response in Algorithm 2 in Figure 5 has a complexity of $O(K^{2})$. Thus, the overall complexity of our proposed resource allocation mechanism is:

$$O\left(K\left(C\log\left(C\right)+\log\left(K\right)+K\right)+\left\lceil \frac{\min(K,S^{n})}{C}\right\rceil C^{2}\right).$$

## 5. Performance Evaluation

We present representative simulation results for the proposed scheme and compare its performances with the state-of-the-art works and the optimal method. We use ns-3 [17] as the simulation tool and develop corresponding IEEE 802.22 modules for channels, ports, users, etc. We deploy different network entities, namely BSs, CPEs, PUs, etc., in our simulation environment system and apply different resource allocation algorithms to study the comparative performances. Designing all the network entities by following the IEEE 802.22 standard with the listed network parameters in Table 2 conforms with the homogeneity of the nodes and links.

### 5.1. Environment Setup

We consider an area of 75 km × 86 km, in which nine IEEE 802.22 overlapping network cells are deployed. We deploy a random number of CPEs ranging from 20–100 across the entire region using a uniform distribution. Moreover, there are random numbers of PUs placed across the area. We implement our proposed algorithm, DSPG, in each BS and CPE, which are designed to sense the environment and share the data with their associated BSs. Simulation parameters are summarized in Table 2.

For comparing the relative performances, we also implement two other state-of-the-art works, namely QBC [14] and the bisection method for power allocation and greedy subchannel allocation (BPGS) [24], in each BS. While QBC is based on centralized spectrum manager, BPGS is a distributed solution for a similar network environment. Both of these works are slightly modified for our network model. In the implementation of QBC, the queue size for BSs is dynamically calculated using their proposed method; therefore, there is no need for an explicit congestion control algorithm. BPGS is designed for downlink subchannel allocation. Therefore, apart from the distance between the transceivers and path loss model, no changes are made for its implementation.

We compute the results for the optimal resource allocation in a cloud-based IAAS server having a 2.6-GHz quad core processor and 2 GB of RAM powered by a 32-bit Ubuntu 14.04 LTS. The optimizer is modeled using Convex.jl [50] tools. In this platform, it requires 400 s–450 s on average to compute the optimal result for the parameters presented in Table 2. By contrast, the non-optimal solutions DSPG, BPGS and QBC reach their convergence point in at most 10 s. For convenience in the comparison, all the parameters presented here for the optimal result are taken after the completion of the total computation, and they are only presented for the comparison of the achievable data rate (average per CPE and network).

### 5.2. Performance Metrics

We compare the performances of the resource allocation algorithms, that is DSPG, QBC [14] and BPGS [24], based on the following metrics

– Data rate: The data rate is the achievable bit-rate value given in Equation (3), which is calculated using Shannon’s capacity formula [49]. Because, we only consider the downlink resource allocation, we measure the data rate values at the CPEs.
– Tenth percentile data rate: Fairness is orchestrated by measuring the 10th percentile data rate of the network cells. This means that more than 10% of the CPEs have data rates higher than the measurement, presented in the associated graphical representations.
– Convergence cost: The convergence cost is measured by the number of iterations required per network to reach the NE point. A high convergence cost adds overhead to the algorithm performance and delays the scheduling process.
– Aggregated utility: To measure the optimality of the algorithms, we calculate the aggregated utility values given in Equation (6). For effective comparison of DSPG and BPGS with the optimal value, the utility value is calculated using Equation (18) in each BS separately and aggregated later. Then, we take the percentage of the aggregated utility value with respect to the optimal value obtained using Equation (6) under the same network environment constraints.

### 5.3. Experimental Results

To evaluate the performance of our proposed DSPG, we study the performance by varying the number of CPEs in the total network area. We also collect results with varying numbers of PU density to analyze the effects of PUs. As the operation of PUs over the spectrum is random and varied over time, we take the percentage of spectrum usage by PUs at a given time; for example, 60 subchannels with 30% primary incumbent density means that the PUs occupy 30% of the total 60 subchannels at a given time. We also study the effect of the network power budget imposed by the BSs. The network power budget is the amount of transmission power for each BS in all subchannels. We compute the average of the results from 25 simulation runs and present the confidence interval for data points in the graphs.

#### 5.3.1. Impact of CPE Density

In this experiment, we vary the number of CPEs from 20–100 and place them in a network area with a uniform random distribution. We deploy seven BSs each having maximum a 46-dBm power budget and considering 60 subchannels with 30% primary incumbent density.

**Figure 6:**
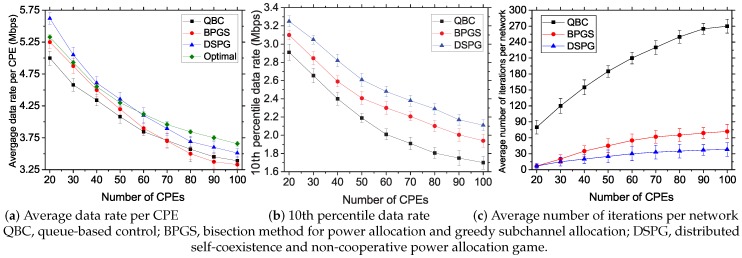
Impact of CPE density on the performance of the studied resource allocation systems.

The curves in Figure 6a depict the average data rate achieved by each CPE in Mbps. The results show that the average data rate significantly decreases for all the studied resource allocation mechanisms with increasing number of CPEs. Our network resources (number of subchannels and power budget for each BS) are limited, and thus, the increasing number of CPEs intensifies the interference, which eventually reduces the data rate. However, the proposed DSPG offers a higher data rate compared to those of QBC and BPGS. This is because DSPG increases the spatial channel utilization by allowing transmissions from coexisting networks at optimal powers on the least interfering subchannels, as opposed to deferring transmissions from neighboring nodes on a subchannel in QBC and BPGS. Prioritizing the performance of CPEs by using a CPE metric helps DSPG achieve a better average data rate than the optimal result at a lower number of CPEs. Although this affects the overall network data rate, a moderate QoS level across all CPEs is achieved by using DSPG.

In Figure 6b, we measure the 10th percentile data rate of the studied systems with varying CPEs. This helps us to compare the degree of fairness achieved by the CPEs throughout the network. In terms of fairness, the proposed DSPG system significantly outperforms both state-of-the-art works. The CPE metric, defined in Equation (16), helps DSPG to achieve a high data rate fairness by taking into consideration the historical channel usage behavior of different CPEs. Moreover, Figure 6b clearly shows QBC’s approach for higher importance on the data rate value rather than compromising it for fairness. For BPGS, the value of the fairness factor ζ is taken as 0.4. However, increasing the ζ value increases the 10th percentile data rate value with the cost of reducing the average data rate obtained in Figure 6a. It also affects the efficiency of the algorithm given in BPGS. As a summary, our DSPG offers moderate fairness without loss of efficiency and average data rate.

The graphs in Figure 6c show the iteration counts to reach the equilibrium point by the studied systems. The proposed DSPG requires much fewer iterations to reach the NE point, when compared to both state-of-the-art works. DSPG’s distributed solution approach and lower dependency on neighboring BSs of the network, as well as on the backhaul network help it to reduce the iteration count by water-filling the power level eventually, in contrast to the step size approximations in conventional approaches. BPGS’s overhead comes from the computation of several parameters and sharing between BSs to estimate the final results. QBC’s B&B algorithm creates more branches to fathom, which leads to an increased number of iterations. Furthermore, the iteration count is dependent on the degree of the generalized optimization algorithm, which increases the count value.

#### 5.3.2. Impact of PU Density on the Network

Here, we discuss the performance results on varying primary incumbent densities. Note that only the PU transmitters will create interference to our CPEs. Therefore, we control the transmission from the PUs such that they occupy 30% of the total 60 subchannels at a given time. The occupancy percentage is varied from 10%–70%, which resulted in 54–18 vacant available subchannels at a given time. Other parameters are kept constant: number of BSs = 7, number of CPEs = 80, total number of subchannels = 60 and power budget of a BS = 46 dBm.

**Figure 7:**
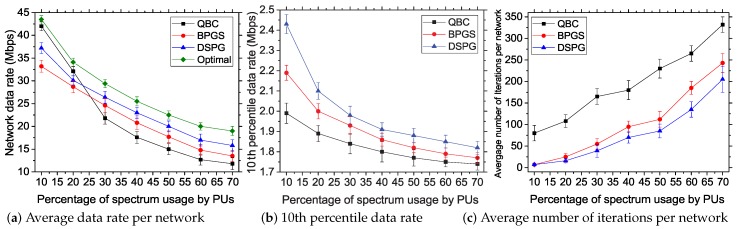
Impact of PU density on the performance of the studied resource allocation systems.

In Figure 7a, we quantify the total user data rate per network observed during the simulation period. Here, we take the total data rate instead of the average because the increment of spectrum usage increases the interference to nearby CPEs. BPGS assigns a subchannel to a CPE with a low transmission power even if it resides in a high interference region. However, DSPG ignores the CPEs with high interference values because transmissions toward that CPE from BS will further increase the interference to the primary receivers, as well. Again, QBC uses different interference thresholds in assigning subchannels. Therefore, the rate of change in the average data rate for different spectrum usage parameters is different, and it does not provide the required information for the network. On the other hand, the total data rate provides a proper insight into the data rate achieved in the CPEs. We can observe that for less than 30% spectrum usage values, BPGS provides a better total data rate, which eventually reduces with the increment of spectrum usage. Not changing the channel assignment and waiting for the distress signal from the PU affect the aggregated data rate. DSPG changes the channel assignment upon receiving the sensing results, which helps to maintain a better data rate during high densities of PUs in the spectrum bands. Although QBC achieves a network data rate as great as the optimal result for low PU densities, its performance degrades over high PU densities as it does not incorporate PU interferences directly in its objective function.

We can observe this effect more clearly on the 10th percentile data rate in Figure 7b. Even if the aggregated data rate is better in BPGS for lower PU densities in the spectrum bands, the 10th percentile data rate is lower than that achieved in DSPG. BPGS can achieve better fairness compared to DSPG by compromising the network data rate (ζ>0.4). The overall result suggests that our proposed DSPG offers a better data rate in terms of fairness by increasing PU spectrum usage percentages.

In Figure 7c, we show the convergence cost in terms of the number of iterations per network. In this case, for low spectrum usage by PUs, DSPG and BPGS require almost the same number of iterations, while QBC requires a larger iteration number to reach the convergence point. The iteration count increases more rapidly in all cases for the increment of spectrum usage, as the network becomes more dynamic for higher spectrum usage values.

#### 5.3.3. Impact of Number of BSs

Here, we analyze the results with varying network setups by deploying different numbers of BSs using a honeycomb model of networking with a total area of (75×86) km^2^, (75×65) km^2^, (75×44) km^2^ and (45×44) km^2^ for 9, 7, 5,= and 3 BS network setups, respectively. Other parameters are kept constant: number of CPEs = 80, total number of subchannels = 60 with 30% PU density and power budget for a BS = 46 dBm.

**Figure 8:**
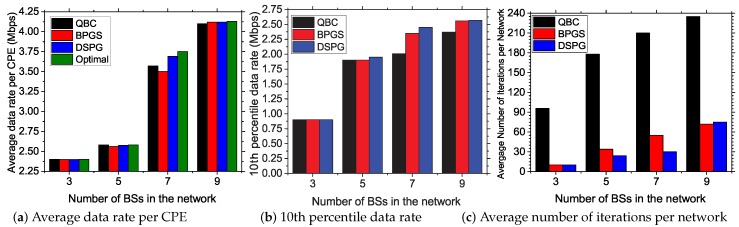
Impact of the number of BSs in the network on the performance of the studied resource allocation systems.

In Figure 8a, we present the average data rate per CPE in Mbps for different network setups. In this case, we observe that all three approaches achieve almost the same values of the optimal average data rate. A network with 3 BSs shows a low data rate of 2.375 Mbps on average. For this case, most of the CPEs cannot acquire a subchannel because of the limited network resources. The CPEs acquiring the vacant subchannels have low data rates owing to high interference from neighboring networks. For a network setup with increased number of BSs, the available network resources are increased, and thus, the probability of BS association increases for each CPE, which helps to achieve the data rate exponentially, as shown in Figure 8a.

In Figure 8b, the 10th percentile data rate is shown for different network setups. In this case, the performance of all approaches is almost the same for small BS setups. However, for large number of BSs, QBC suffers owing to its thresholding of the interference values, which imposes a low transmission power toward the CPEs. Figure 8c shows the convergence cost for different network setups in terms of the average iteration count per network. QBC suffers most for all network setups. Again, its B&B-based algorithm resulted in higher iteration cost. Our proposed DSPG offers a lower iteration cost owing to its autonomous calculation and lower dependency on neighboring BSs and the backhaul network.

#### 5.3.4. Network Power Budget Estimation

In this experiment, we estimate the effects of the power budget on the data rate and allocated power level for different α values. We provide an analysis of α values by comparing SINR and power for each subchannel. Then, an estimation of the average data rate per network is performed for different power budgets.

**Figure 9:**
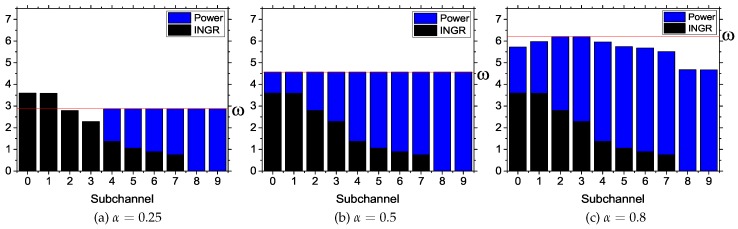
Effect of α on the water level and allocated power values.

We show the changes in water level with different α values in Figure 9. The network parameters are 7 BS, 60 CPEs and 50 subchannels with 80% usage of the spectrum by PUs. Here, the estimation is performed for a BS in different subchannels with a total power budget of 40 W. The interference plus noise-to-gain ratio (INGR) is the inverse of $\xi_{s,k}^{n}$, and ω represents the water level. The subchannels are sorted in decreasing order of their associated CPE INGR. In Figure 9a, for α=0.25, the total water level is 2.875 W. Here, Subchannels 0 and 1 have more INGR than the water level. Therefore, DSPG does not assign any power to them. For Subchannels 2 and 3, although the INGR is lower than the water level, their achieved SINR value does not reach their minimum required SINR $\left(\gamma_{s}\right)$ level. For low water levels, only 13.1766 W is assigned among the rest of the subchannels, and the remaining power is saved or is not allocated. In Figure 9b, for α=0.5, the total water level reaches 4.566 W. In this case, the water level is higher than the INGR level of all subchannels. Therefore, all subchannels are assigned a power value. Here, only 29.31671 W is assigned, and the rest is unallocated. In Figure 9c, for α=0.8, the total water level reaches 6.20333 W. In this case, all 40 W are assigned and distributed to all subchannels. The total allocated power value will not change for α>0.8, because 40 W is the maximum power for a BS, although it increases the water level. Therefore, this experimental result suggests that for high α values, a better data rate and CPE coverage are achieved by maximizing the power usage of the BSs. Decreasing the α value will save power by compromising the data rate and eliminating CPEs with high interference.

**Figure 10:**
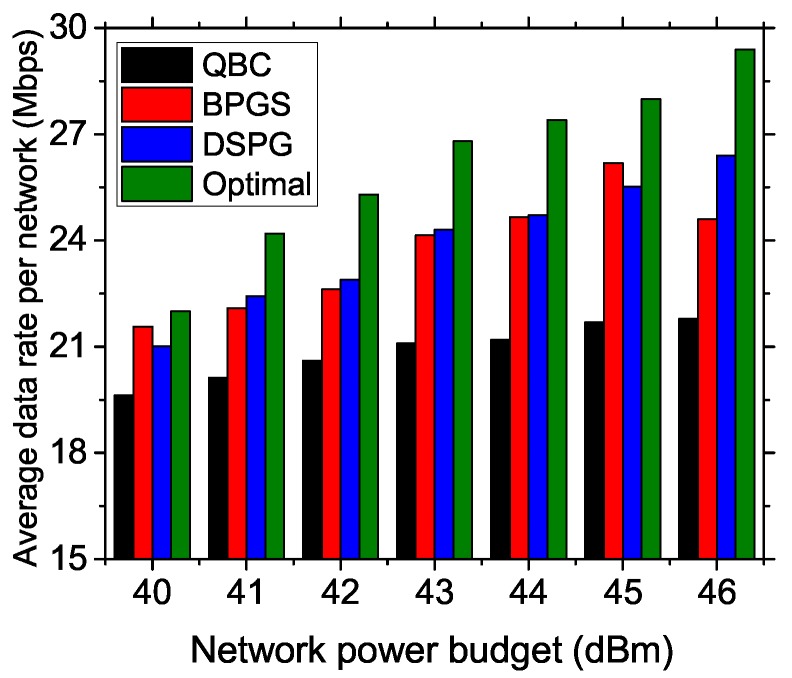
Average data rate vs. power budget of a BS.

In Figure 10, we show the average data rate achieved per network for different power budgets applied at the BSs. The network power budget is varied from 40 dBm–46 dBm. Other network parameters are taken as 7 BSs, 80 CPEs and 60 subchannels with 30% spectrum usage by PUs. Our proposed DSPG has achieved a better data rate per network, as it distributes total power values to at least one CPE; furthermore, interference-free CPEs are getting high data rate values for low transmission power. A comparison with the optimal results suggests that all the approaches are lagging behind in terms of optimal power allocation. However, DSPG has a smaller lag toward the optimal result compared to the other approaches.

#### 5.3.5. Estimation of Aggregated Utility at Different BSs

**Figure 11:**
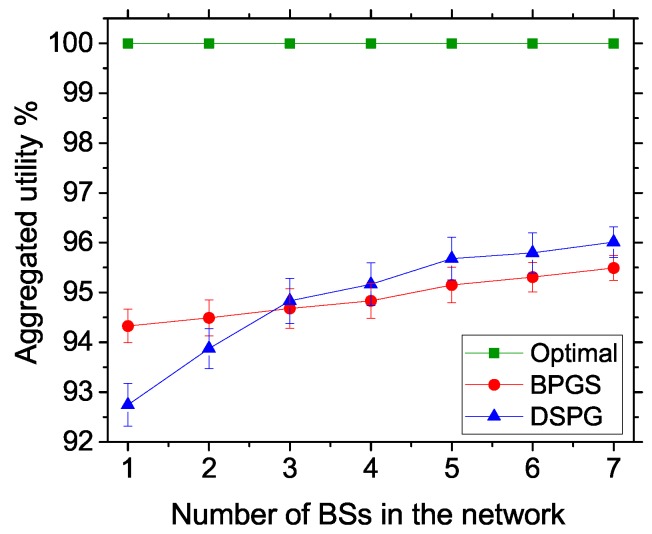
Estimation of aggregated utility for different numbers of BSs.

In Figure 11, we show the utility values for DSPG and BPGS compared to the optimal result. We model the optimization by using Equation (6) with the constraints in Equations (7)–(13) and take the maximized values for different number of BSs (1–7). In the DSPG system, after achieving the NE point, the sum of utility values in Equation (18) is taken for different BSs. Note that with the sum for different networks, Equation (18) is equivalent to Equation (6). We take the percentage of DSPG utility by considering the optimal value as 100%. Note that α=1 is set for convenience of a fair comparison with BPGS. In BPGS, the same approach is taken, except for the modified greedy channel assignment algorithm and bisection method for power allocation. In this case, BPGS shows an almost average performance of up to 95.49% utility gain, whereas DSPG gains up to 96.01% aggregated utility. Note that in both cases, the power values are suboptimal based on the assigned channels. Different greedy approaches are affecting the aggregated utility value in both cases. Therefore, we can conclude that our autonomous approach achieves almost the same performance and, in some cases, a better gain compared to a non-autonomous approach.

In summary, the above results suggest that our proposed DSPG system is capable of using BS power for various network conditions more practically. Our formulated objective function provides an insight into using power on selected subchannels in such a way that the interference is minimized and the network data rate is maximized. This helps us to design a greedy solution that achieves significant performance improvements for our network environment.

## 6. Conclusions

This study explored downlink resource allocation strategies for a cognitive wireless sensor network. A mixed-integer non-linear programming problem was developed to maximize the network data rate while keeping the interferences among the cells at a minimum. Owing to the NP-hardness of the optimization problem, we divided it into two subproblems: greedy subchannel assignment and game-theoretic optimal power allocation. Our new metric for quantifying the suitability of a subchannel for a CPE in terms of channel gain and interference(s) greatly facilitated the proposed greedy subchannel assignment algorithm and game-theoretic power allocation scheme in achieving better performances. The best response for each subchannel was calculated through an iterative approach. The proposed method was able to complete the resource allocation without any cooperation from PUs, and BSs were operated independently. We provided theoretical proofs of the existence and uniqueness of the NE in the proposed game model. The performance results proved the efficacy of the proposed system compared to state-of-the-art works. Our simulation results reflected that a near optimal resource allocation is possible by minimizing power consumption in a dynamic network environment without any direct cooperation between the network entities. We have only analyzed the downlink resource allocation problem for the IEEE 802.22 networks; a slight modification in utility function with proper interference modeling can extend our work for the uplink resource allocation, as well.

## Figures and Tables

**Table 1:** Major notations.

Symbol	Definitions
$S_{c}^{n}$	Set of sessions assigned by BS n∈N
	to CPE c∈C
$S^{n}$	Set of sessions assigned by BS n∈N,
	where $S^{n}={⋃}_{c\in C}S_{c}^{n}$
$S_{c}$	Set of sessions initiated by CPE c∈C
S	Set of all sessions in the total vicinity area,
	where $S={⋃}_{c\in C}S_{c}$
D	${\left\{d_{s,k}\right\}}_{|S|\times K}$, where $d_{s,k}$ is a binary indicator
	having ‘1’ if downlink subchannel k∈K is
	assigned to session s∈S and ‘0’ otherwise
$h_{s,k}^{n}$	Channel gain from BS n∈N,s∈S,k∈K
$p_{k}^{n}$	Transmission power of BS *n* in subchannel k∈K
$P^{n}$	${\left\{p_{k}^{n}\right\}}_{K\times 1}$ Transmission power vector of BS n∈N
$p_{\max}^{n}$	Maximum power level of a BS n∈N
P	Power allocation matrix $\left\{P^{1},P^{2},\cdots ,P^{N}\right\}$
$\eta_{0}$	Average Gaussian noise power
$\gamma_{s}$	Minimum SINR of session s∈S to achieve
	a certain BER
$\sigma_{s,k}^{n}$	Calculated SINR of session $s\in S^{n}$ in k∈K
$\theta_{s}$	Quality-of-service requirement for session s∈S
$q_{s,k}^{n}$	Data rate of session $s\in S^{n}$ in k∈K
$I_{s,k}^{n}$	Total interference inflicted by session
	$s\in S^{n}$ in k∈K
$I_{c,k}^{PU}$	PU interference inflicted by CPE c∈C in
	subchannel k∈K

**Table 2:** ns-3 implementation parameters.

Parameter	Value
Node mobility	Stationary
Area	75 km × 86 km
Operational frequency	470 MHz–608 MHz
Bandwidth	6 MHz
Duplexing method	TDD
Modulation type	16-QAM
Coding rate	1/2
Path loss exponent	2
Transmission range	25 km–30 km
BS Sensing range	50 km–75 km
CPE Sensing range	10 km–25 km
Max. transmission power	46 dBm
α	0.8
ω	0.001
$T^{n}$	min{10,numberofprevioussteps}
Propagation loss model	Friis propagation loss model [49]
Noise model	(AWGN)

## Appendix A. Proof of Proposition 1

**Proof.** After the completion of sub-channel assignment, we can only consider the sessions *s* where $d_{s,k}=1$. Thus, we can eliminate the iteration over the sessions from the utility function in Equation (18), and we get,

$$\begin{array}{cc} U^{n}\left(P\right) & =\sum_{k\in K}R_{s,k}^{n}\\ & =\sum_{k\in K}\left\{\alpha \frac{q_{s,k}^{n}}{Q_{s,k}^{n}}-\left(1-\alpha \right)\frac{p_{k}^{n}}{p_{\max}^{n}}\right\}\\ & =\sum_{k\in K}\left\{\alpha \frac{B\log_{2}\left(1+\xi_{s,k}^{n}p_{k}^{n}\right)}{Q_{s,k}^{n}}-\left(1-\alpha \right)\frac{p_{k}^{n}}{p_{\max}^{n}}\right\}\\ & =\sum_{k\in K}\left\{\phi_{1,k}^{n}B\log_{2}\left(1+\xi_{s,k}^{n}p_{k}^{n}\right)-\phi_{2}^{n}p_{k}^{n}\right\} \end{array}$$

where,

$$\begin{array}{c} \phi_{1,k}^{n}=\frac{\alpha }{Q_{s,k}^{n}},\;\phi_{2}^{n}=\frac{\left(1-\alpha \right)}{p_{\max}^{n}},\\ \xi_{s,k}^{n}=\frac{h_{s,k}^{n}}{\sum_{a=1,a\neq N}^{n}h_{s,k}^{a}p_{k}^{a}+I_{c,k}^{PU}+\eta_{0}}. \end{array}$$

Therefore, the Lagrangian can be written as,

$$\begin{array}{cc} L(P^{n},\lambda_{1}^{n},\lambda_{2}^{n},\lambda_{3}^{n})= & \sum_{k\in K}\phi_{2}^{n}p_{k}^{n}-\phi_{1,k}^{n}B\log_{2}\left(1+\xi_{s,k}^{n}p_{k}^{n}\right)\\ & -{\left(\lambda_{1}^{n}\right)}^{T}p^{n}+\lambda_{2}^{n}\left(\sum_{k\in K}p_{k}^{n}-p_{\max}^{n}\right)\\ & -\sum_{k\in K}\lambda_{3,k}^{n}B\log_{2}\left(1+\xi_{s,k}^{n}p_{k}^{n}\right)-{\left(\lambda_{3}^{n}\right)}^{T}\theta \end{array}$$

where $\lambda_{1}^{n}\in R^{K\times 1},\lambda_{2}^{n}\in R,\lambda_{3}^{n}\in R^{K\times 1}$ are Lagrange multipliers. Now, we get,

$$\begin{array}{cc} \frac{\partial L}{\partial p_{k}^{n}} & =\phi_{2}^{n}-\lambda_{1,k}^{n}+\lambda_{2}^{n}-\left(\phi_{1,k}^{n}+\lambda_{3,k}^{n}\right)B\log_{2}^{e}\frac{\xi_{s,k}^{n}}{1+\xi_{s,k}^{n}p_{k}^{n}} \end{array}$$

By applying Karush–Kuhn–Tucker (KKT) [43] conditions,

$$\begin{array}{cc} & \frac{\partial L}{\partial p_{k}^{n}}=0\\ \Rightarrow \; & \phi_{2}^{n}-\lambda_{1,k}^{n}+\lambda_{2}^{n}=\left(\phi_{1,k}^{n}+\lambda_{3,k}^{n}\right)B\log_{2}^{e}\frac{\xi_{s,k}^{n}}{1+\xi_{s,k}^{n}p_{k}^{n*}}\\ \Rightarrow \; & \frac{\xi_{s,k}^{n}}{1+\xi_{s,k}^{n}p_{k}^{n*}}=\frac{\phi_{2}^{n}-\lambda_{1,k}^{n}+\lambda_{2}^{n}}{\left(\phi_{1,k}^{n}+\lambda_{3,k}^{n}\right)B\log_{2}^{e}} \end{array}$$

(A1) $$\Rightarrow \;p_{k}^{n*}=\frac{\left(\phi_{1,k}^{n}+\lambda_{3,k}^{n}\right)B\log_{2}^{e}}{\phi_{2}^{n}-\lambda_{1,k}^{n}+\lambda_{2}^{n}}-\frac{1}{\xi_{s,k}^{n}}$$

Complementary slackness:

(A2) $$\begin{array}{cc} \lambda_{1,k}^{n*}p_{k}^{n*}=0, & \forall k\in K \end{array}$$

(A3) $$\begin{array}{c} \lambda_{2}^{n*}\left(\sum_{k\in K}p_{k}^{n*}-p_{\max}^{n}\right)=0, \end{array}$$

(A4) $$\begin{array}{cc} \lambda_{3,k}^{n*}B\log_{2}\left(1+\xi_{s,k}^{n}p_{k}^{n*}\right)-\lambda_{3,k}^{n*}\theta_{s}=0, & \forall k\in K \end{array}$$

Primal dual constraints:

(A5) $$\begin{array}{cc} p_{k}^{n*}\geq 0, & \forall k\in K \end{array}$$

(A6) $$\begin{array}{cc} B\log_{2}\left(1+\xi_{s,k}^{n}p_{k}^{n*}\right)\geq \theta_{s}=0, & \forall k\in K \end{array}$$

(A7) $$\begin{array}{cc} \sum_{k\in K}p_{k}^{n*}=p_{\max}^{n} & \end{array}$$

(A8) $$\begin{array}{c} \lambda_{1}^{n*},\lambda_{3}^{n*}⪰0 \end{array}$$

(A9) $$\begin{array}{cc} \lambda_{2}^{n*}\geq 0 & \end{array}$$

Using the KKT theorem, we have the best response of BS n∈N in channel k∈K that satisfy (A2)–(A9):

$$\Rightarrow p_{k}^{n*}={\left[\frac{\left(\phi_{1,k}^{n}+\lambda_{3,k}^{n*}\right)B\log_{2}^{e}}{\phi_{2}^{n}-\lambda_{1,k}^{n*}+\lambda_{2}^{n*}}-\frac{1}{\xi_{s,k}^{n}}\right]}^{+}$$

By putting $p_{k}^{n*}$ in Equation (A7), we get,

$$\sum_{k\in K}{\left[\frac{\left(\phi_{1,k}^{n}+\lambda_{3,k}^{n*}\right)B\log_{2}^{e}}{\phi_{2}^{n}-\lambda_{1,k}^{n*}+\lambda_{2}^{n*}}-\frac{1}{\xi_{s,k}^{n}}\right]}^{+}=p_{\max}^{n},$$

which concludes Proposition 1. ☐

## Appendix B. Proof of Theorem 2

**Proof.** From the best response function in Equation (24), we have,

$${\mathrm{BR}}_{k}^{n}\left(p_{k}^{-n}\right)=p_{k}^{n*}={\left[\frac{\left(\phi_{1,k}^{n}+\lambda_{3,k}^{n*}\right)B\log_{2}^{e}}{\phi_{2}^{n}-\lambda_{1,k}^{n*}+\lambda_{2}^{n*}}-\frac{1}{\xi_{k}^{n}}\right]}^{+},\forall k\in K$$

(A10) $$p_{k}^{n*}={\left[\frac{\left(\phi_{1,k}^{n}+\lambda_{3,k}^{n*}\right)B\log_{2}^{e}}{\phi_{2}^{n}-\lambda_{1,k}^{n*}+\lambda_{2}^{n*}}-\frac{\sum_{a=1,a\neq N}^{n}h_{s,k}^{a}p_{k}^{a}+I_{c,k}^{PU}+\eta_{0}}{h_{s,k}^{n}}\right]}^{+}$$

The existence of equilibrium was already proven in Theorem 1. By showing that Equation (A10) is a contraction mapping, we can prove jointly the uniqueness and convergence of the equilibrium. Let us revisit the definition of contraction mapping theorem [47] as follows: Contraction mapping theorem: Let a complete metric space *M* and a mapping f:M→M. For any constant 0≤k<1 and ∀u,v∈M, if d(f(u),f(v))≤kd(u,v); then, *f* is called a contraction. Then, *f* has a unique fixed point $u^{*}\in M$, and the updated point u→f(u) eventually converges to the unique fixed point. Let Mbe the Euclidean space and d(.) be the induced distance function by a vector norm. Then, we have,

$$\begin{array}{ccc} d(f(u),f(v)) & = & \left||f(u)-f(v)|\right|\\ & \leq & \left|\right|\frac{\partial f}{\partial x}\left||||\left(u-v\right)|\right|=\left|\right|\frac{\partial f}{\partial x}\left|\right|d\left(u,v\right). \end{array}$$

The matrix norm is induced by the vector norm, and the property of the matrix norm brings the inequality. Now, if we can prove that the Jacobian $\left|\right|\frac{\partial f}{\partial x}\left|\right|<1-\epsilon$ everywhere for some positive ϵ, we let k=1−ϵ<1, conforming the contraction mapping theorem. Since, ϵ is arbitrarily small, we ignore it in the following derivations. The Jacobian J for Equation (A10) is defined as,

(A11) $$J_{na}=\frac{\partial p_{k}^{n}}{\partial p_{k}^{a}}.$$

Now, if n=a, then $J_{na}=0$ and, if n≠a, then:

$$\frac{\partial (p_{k}^{n})}{\partial p_{k}^{a}}=\frac{B\log_{2}^{e}}{\phi_{2}^{n}-\lambda_{1,k}^{n*}+\lambda_{2}^{n*}}\frac{\partial }{\partial p_{k}^{n}}\left(\phi_{1,k}^{n}\right)-\frac{h_{k}^{a}}{h_{k}^{n}}.$$

It is straightforward that,

$$\frac{\partial }{\partial p_{k}^{a}}\left(\phi_{1,k}^{n}\right)=\frac{\alpha \log_{2}^{e}p_{\max}{\left(\xi_{k}^{n}\right)}^{2}}{B{\left(\log_{2}\left(1+\xi_{k}^{n}p_{\max}^{n}\right)\right)}^{2}\left(1+\xi_{k}^{n}p_{\max}^{n}\right)}\times \frac{h_{k}^{a}}{h_{k}^{n}}.$$

Therefore,

$$\frac{\partial (p_{k}^{n})}{\partial p_{k}^{a}}=\frac{h_{k}^{a}}{h_{k}^{n}}X_{k}^{n},$$

where,

$$X_{k}^{n}=\frac{\alpha p_{\max}^{n}{\left(\xi_{k}^{n}\right)}^{2}{\left(\log_{2}^{e}\right)}^{2}}{\left(\phi_{2}^{n}-\lambda_{1,k}^{n*}+\lambda_{2}^{n*}\right){\left(\log_{2}\left(1+\xi_{k}^{n}p_{\max}^{n}\right)\right)}^{2}\left(1+\xi_{k}^{n}p_{\max}^{n}\right)}-1;$$

it then follows that,

$${\left||J|\right|}_{\infty }=\max_{n}\left\{\frac{|X_{k}^{n}|}{h_{k}^{n}}\sum_{a\neq n}h_{k}^{a}\right\},\forall k\in K.$$

By the assumption in this theorem, we can conclude that $\left|\right|J_{\infty }\left|\right|<1$. Hence, Equation (A10) is a contraction mapping, and both uniqueness and global convergence toward equilibrium are guaranteed [47]. ☐
